# Macroscopic optical physiological parameters correlate with microscopic proliferation and vessel area breast cancer signatures

**DOI:** 10.1186/s13058-015-0578-z

**Published:** 2015-05-27

**Authors:** So Hyun Chung, Michael D. Feldman, Daniel Martinez, Helen Kim, Mary E. Putt, David R. Busch, Julia Tchou, Brian J. Czerniecki, Mitchell D. Schnall, Mark A. Rosen, Angela DeMichele, Arjun G. Yodh, Regine Choe

**Affiliations:** 1 Department of Physics and Astronomy, University of Pennsylvania, 209 S. 33rd St., Philadelphia, PA 19104 USA; 2 Department of Pathology and Laboratory Medicine, The Hospital of the University of Pennsylvania, 3400 Spruce Street, Philadelphia, PA 19104 USA; 3 Pathology Core Laboratory, The Children’s Hospital of Philadelphia, 3615 Civic Center Boulevard, Philadelphia, PA 19104 USA; 4 Department of Biostatistics and Epidemiology, University of Pennsylvania, 423 Guardian Drive, Philadelphia, PA 19104 USA; 5 Division of Neurology, The Children’s Hospital of Philadelphia, 3615 Civic Center Boulevard, Philadelphia, PA 19104 USA; 6 Department of Surgery, The Hospital of the University of Pennsylvania, 3400 Spruce Street, Philadelphia, PA 19104 USA; 7 Department of Radiology, The Hospital of the University of Pennsylvania, 3400 Spruce Street, Philadelphia, PA 19104 USA; 8 Department of Medicine, The Hospital of the University of Pennsylvania, 3400 Spruce Street, Philadelphia, PA 19104 USA; 9 Department of Biomedical Engineering, University of Rochester, 209 Goergen Hall, P.O. Box 270168, Rochester, NY 14627 USA

## Abstract

**Introduction:**

Non-invasive diffuse optical tomography (DOT) and diffuse correlation spectroscopy (DCS) can detect and characterize breast cancer and predict tumor responses to neoadjuvant chemotherapy, even in patients with radiographically dense breasts. However, the relationship between measured optical parameters and pathological biomarker information needs to be further studied to connect information from optics to traditional clinical cancer biology. Thus we investigate how optically measured physiological parameters in malignant tumors such as oxy-, deoxy-hemoglobin concentration, tissue blood oxygenation, and metabolic rate of oxygen correlate with microscopic histopathological biomarkers from the same malignant tumors, e.g., Ki67 proliferation markers, CD34 stained vasculature markers and nuclear morphology.

**Methods:**

In this pilot study, we investigate correlations of macroscopic physiological parameters of malignant tumors measured by diffuse optical technologies with microscopic histopathological biomarkers of the same tumors, i.e., the Ki67 proliferation marker, the CD34 stained vascular properties marker, and nuclear morphology.

**Results:**

The tumor-to-normal relative ratio of Ki67-positive nuclei is positively correlated with DOT-measured relative tissue blood oxygen saturation (R = 0.89, *p*-value: 0.001), and lower tumor-to-normal deoxy-hemoglobin concentration is associated with higher expression level of Ki67 nuclei (*p*-value: 0.01). In a subset of the Ki67-negative group (defined by the 15 % threshold), an inverse correlation between Ki67 expression level and mammary metabolic rate of oxygen was observed (R = −0.95, *p*-value: 0.014). Further, CD34 stained mean-vessel-area in tumor is positively correlated with tumor-to-normal total-hemoglobin and oxy-hemoglobin concentration. Finally, we find that cell nuclei tend to have more elongated shapes in less oxygenated DOT-measured environments.

**Conclusions:**

Collectively, the pilot data are consistent with the notion that increased blood is supplied to breast cancers, and it also suggests that less conversion of oxy- to deoxy-hemoglobin occurs in more proliferative cancers. Overall, the observations corroborate expectations that macroscopic measurements of breast cancer physiology using DOT and DCS can reveal microscopic pathological properties of breast cancer and hold potential to complement pathological biomarker information.

**Electronic supplementary material:**

The online version of this article (doi:10.1186/s13058-015-0578-z) contains supplementary material, which is available to authorized users.

## Introduction

Diffuse optical imaging and spectroscopy measure deep tissue physiology *in vivo* using low-power non-ionizing light, and in recent years, cancer research with diffuse optics has become more quantitative [1–3]. Several features of diffuse optics make the technology appealing for use in patients with breast cancer, including technical simplicity, portability, soft compression of the breast, non-invasiveness and the cost. Furthermore, the technology has been demonstrated to detect/characterize breast tissue properties regardless of patient age and breast radiographic density [4–8], and it can be used to monitor patient responses to therapy continuously at the bedside [9–14].

The primary endogenous physiological information derived from diffuse optical measurements is oxyhemoglobin, deoxyhemoglobin and total hemoglobin concentration, tissue blood oxygenation [1, 3, 5–8, 14, 15], blood flow [13, 16], and water and lipid concentration [1, 3, 5–8, 14, 15]. More recently, tissue temperature, the binding state of water [7, 10, 17–19] and collagen [20, 21] are proving to be interesting biomarkers. Several groups have reported contrast between breast cancer, benign lesions, and normal tissues based on these physiological parameters [6, 22–24], and tumor responses to neoadjuvant chemotherapy (NAC) have been monitored successfully [9, 11, 13, 14, 25–27]. Some of these responses predict complete versus non-complete pathologically determined response among patients during the early stages of NAC [9, 28, 29] and even before therapy [30].

In this paper we examine how macroscopic diffuse optical parameters are related to microscopic pathology information that clinicians typically employ for treatment strategy decisions. In clinical practice, tumor samples are characterized based on microscopic analyses of immunohistologically stained biopsy specimens. For example, Ki67 expression level in cell nuclei is often assessed to quantify proliferation of cancer cells [31, 32], and CD34 staining is used for quantifying endothelial cells of micro-vessels in order to assess angiogenesis in tumors [33]. A few studies have compared microscopic markers to the parameters derived from diffuse optical images [30, 34–39]. Total hemoglobin concentration in breast cancer, for example, has been positively correlated with vascular properties such as micro-vessel density [37–39]. Although correlations between Ki67 proliferation marker expression level and diffuse optically measured physiological parameters have not been reported, several positron emission tomography (PET) studies have found correlation between Ki67 cancer proliferation level and fluorodeoxyglucose (FDG) metabolism [40–42], but a different study reported no correlation between Ki67 and ^18^F-FDG uptake, and a marginal correlation between Ki67 expression level and tumor-to-background ratio of the uptake of the hypoxia-avid compound ^18^F-labeled fluoromisonidazole (^18^F-FMISO) [43]. The authors of the latter paper concluded that their observations might be due to alteration of glucose metabolism in cancer that prefers aerobic glycolysis, a phenomenon known as the Warburg effect [44]. In the work of Cochet et al. [41], no significant correlation was found between standardized uptake of ^18^F-FDG and endothelial markers (CD34 and CD105). Note also, tumor blood flow indices defined by these authors correlated positively with the expression of CD34 and CD105 and with the expression of Ki67 [41].

Our pilot study provides a more extensive exploration of the potential connections between tissue parameters obtained from diffuse optical tomography (DOT) and diffuse correlation spectroscopy (DCS), and standard histopathological biomarkers derived from the same patient tissues. In previous research we demonstrated that the tumor-to-normal ratio of a variety of parameters in three-dimensional (3-D) DOT images can differentiate benign from malignant breast lesions [6]. Here we focus on malignant tumor properties. Specifically, we investigated how DOT-based physiological parameters in malignant tumors, such as oxyhemoglogin, deoxyhemoglobin concentrations, tissue blood oxygenation, and tumor-to-normal ratio of the mammary metabolic rate of oxygen (rMMRO_2_) (derived from hemoglobin concentration and DCS blood flow data) correlate with microscopic histopathological biomarkers from the same malignant tumors, i.e., with the Ki67 proliferation marker, the CD34 stained vasculature marker, nuclear morphology and with hormonal receptor status of breast cancer.

## Methods

### Subjects

The University of Pennsylvania Institutional Review Board approved our measurement protocol. Written informed consent was obtained from each subject for the diffuse optical measurements and for publishing the data. For this retrospective study, written informed consent was not required for retrieval of specimens routinely stored in the tissue bank. From 37 subjects with cancer analyzed with DOT in our previous publications [6, 16], corresponding pathology slides for 21 subjects were available for additional staining of Ki67 and CD34. Specimens from the remaining subjects were not stored in the tissue bank from which we retrieved the tissues. Although some samples were not available for additional staining, information on 11 of these subjects was acquired from standard-of-care pathology reports for the correlation study with tumor grade and hormonal/genetic subtypes. Thus, a total 32 subjects with infiltrating ductal carcinoma were studied in this paper.

In the 21 subjects used for Ki67, CD34 and nuclear morphology analysis, DOT measurements were performed at three different times: (1) prior to any biopsy in 9 subjects (DOT measurements were made 5 days before the biopsy on average, i.e., 43 % of the sample); (2) prior to core biopsy in 2 subjects (DOT measurements were made 8 days prior to excision of the entire tumor on average, i.e., 9 %), and (3) ≥2 weeks after the biopsy and prior to excision of the entire tumor in 10 subjects (DOT measurements were made 18 days prior to excision of the entire tumor on average, i.e., 48 %).

For the full sample of 32 subjects, DOT measurements were performed (1) prior to any biopsy in 11 subjects (DOT measurements were made 5 days before the biopsy on average, 34 %); (2) prior to core biopsy in 6 subjects (DOT measurements were made 18 days prior to excision of the entire tumor, 19 %), and (3) ≥2 weeks after the biopsy and prior to excision of the entire tumor in 15 subjects (DOT measurements were made 22 days prior to excision of the entire tumor on average, 47 %). We did not observe any biopsy-induced bruises (black or blue) in any subjects, i.e., the patients' biopsy sites healed sufficiently that no visible signs were apparent. In addition, by comparing optical data obtained before versus after biopsy, Choe et al. [6] explicitly tested whether such biopsies affect tumor optical contrast, and no significant differences were found in that investigation. Therefore, we expect that the DOT patient measurements in the present work were not influenced by the biopsies. Different numbers of subjects were studied for each biomarker for various reasons, as described below (also see Additional file 1: Table S1).

#### Ki67

Samples from 18 out of the 21 subjects initially chosen for Ki67 evaluation were used; two slides lacked sufficient cancer tissue and one slide had uneven thickness (which caused out-of-focus microscopic imaging for this computer-assisted analysis). For the calculation of the tumor-to-normal ratio of Ki67 (rKi67), we only used samples from subjects with Ki67 expression in normal tissues, which corresponded to 9 out of the 18 subjects. These 9 cases were found to be pre-menopausal patients. All 18 subjects were used for the cancer-only analysis; Ki67 expression in confirmed cancer tissues from all patients was available for this analysis. (Note, this number is the numerator of the tumor-to-normal ratio (rKi67) for the patients who had both tumor and normal tissue Ki67 expression.) Samples were available for six subjects only for the comparison study between rMMRO_2_ (see *Diffuse correlation spectroscopy (DCS)*) and relative mammary metabolic rate of oxygen (rMMRO_2_)) and Ki67 expression level, because blood flow data are required to calculate rMMRO_2_, and blood flow was measured by diffuse correlation spectroscopy in only six of the subjects included in this retrospective analysis. Among the six subjects, five had low Ki67 values and belonged to the Ki67-negative group; samples from these subjects were investigated for correlation with rMMRO_2_. Of these six, only two subjects had Ki67 expression in normal tissues and were therefore available for rMMRO_2_ versus rKi67 analysis.

#### CD34

Only the vascular structures of cancer tissues were available for study, because normal vessels were rarely observed adjacent to the biopsied cancer tissues. Generally, normal vessels appear far outside of the entire tumor structure, and therefore normal vessels were seldom observed in the small specimens. Of the 21 subjects, 19 were available for this analysis, because one specimen slide had uneven thickness and did not have sufficient cancer tissue for analysis.

#### Nuclear compactness

Samples from all 21 subjects were available for the nuclear compactness analysis.

#### Other analyses

In addition to the 21 subjects described above, we analyzed samples from an additional 11 subjects for whom DOT images and clinical pathology reports were available. Unfortunately, there were no tissue samples from these subjects for the additional staining described above. These additional subjects were included in some of our analyses to investigate the relationship between DOT physiological parameters, hormonal and genetic features, and breast cancer tumor grade. Among the total of 32 subjects, modified Bloom-Richardson scores were available for 29 subjects, and cancer hormonal status was reported for 31 subjects.

### Diffuse optical tomography (DOT)

**Fig. 1:**
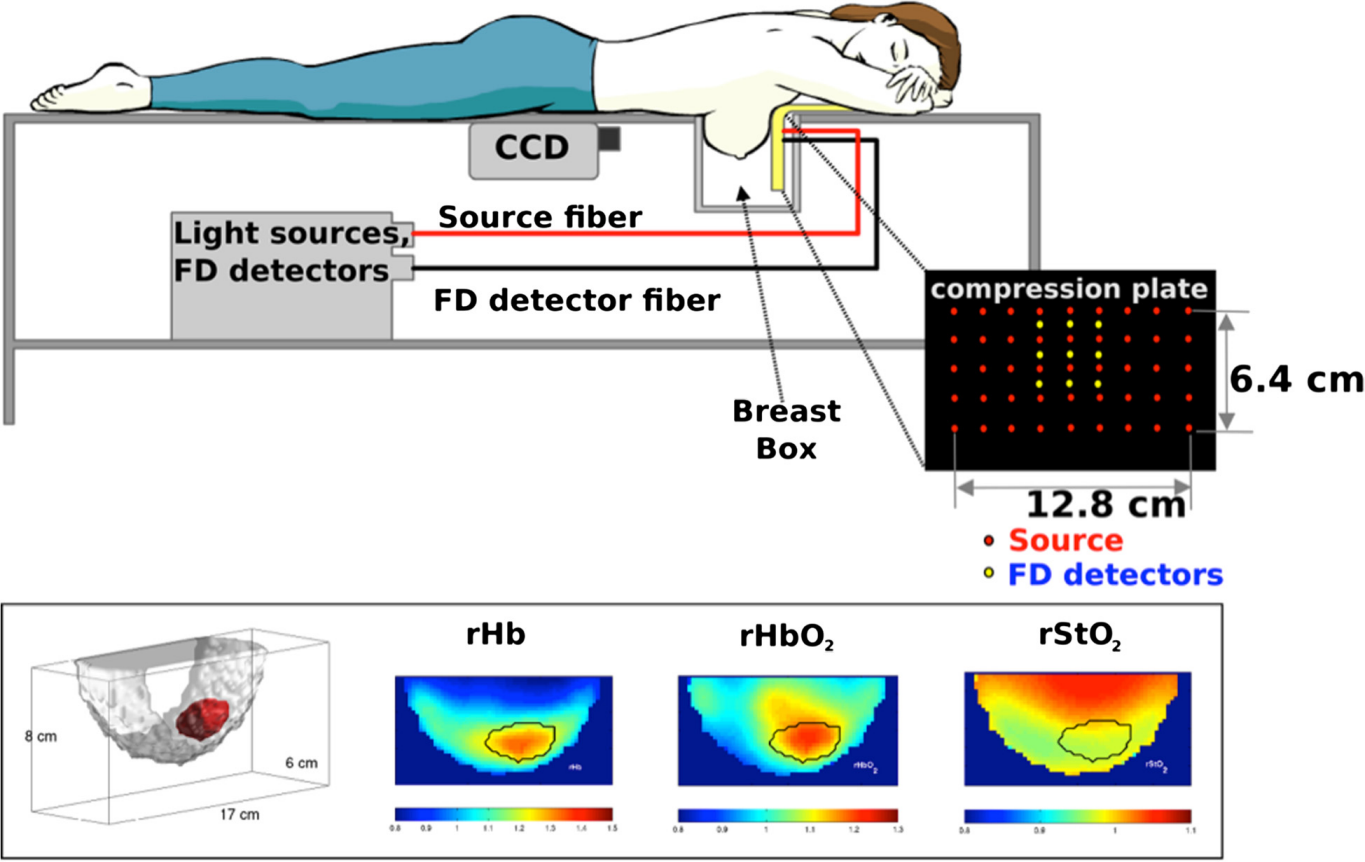
Instrumental setup for the diffuse optical tomography (DOT) system, and DOT images acquired from a 53-year-old woman with a 2.2-cm (longest dimension) invasive ductal carcinoma. *Bottom left* depicts a three-dimensional tumor region (*red*). The images of *rHb*, *rHbO*_*2*_ and *rStO*_*2*_ are for relative (i.e., tumor-to-normal ratio) deoxyhemoglobin and oxyhemoglobin concentration and tissue oxygenation, respectively. *Black solid line* in the images indicates the region identified as tumor. *FD* frequency domain, *CCD* charge-coupled-device camera

Details of the DOT system, measurement procedure and reconstruction algorithm are described in Choe et al. [6]. Briefly, the instrument employed the parallel-plate geometry for source and detector planes; a breast box was utilized wherein breasts were positioned while the subject was lying in a prone position. The breasts were compressed mildly to hold them in a stable position. An outline of the breast surface was obtained by a 16-bit charge-coupled-device (CCD) camera. An Intralipid/ink (Baxter, Deerfield, IL) solution with optical properties similar to breast tissue filled the box to form a background diffuse medium and thereby reduce dynamic-range requirements. Four lasers at 690, 750, 786 and 830 nm were modulated at 70 MHz, and two lasers at 650 and 905 nm were used in continuous-wave (CW) mode. Laser light was delivered to 45 source locations using optical switches shown as red dots in Fig. 1. The source plane also had nine fibers embedded within it (yellow dots in Fig. 1) for detection of modulated light in the remission geometry. These frequency-domain (FD) measurements provided bulk optical properties that served as initial guesses for further reconstruction. CW-domain transmission measurements were made through a transparent window utilizing the CCD camera. The 45 source locations and the CCD camera enabled collection of a very large dataset for reconstruction of 3-D optical images. The diffuse optical data for the images were acquired in 8–12 minutes. Following the subject measurement, a phantom calibration measurement was performed after fully filling the breast box with the same Intralipid/ink solution and covering the top of the box with a silicone slab to mimic the chest wall. A brief description on the reconstruction steps for generating 3-D optical images is included in Additional file 1. Three-dimensional images of oxyhemoglobin, deoxyhemoglobin and total hemoglobin concentrations, tissue oxygenation and reduced-scattering coefficients were obtained.

Determination of tumor boundaries in the optical images has previously been described [6]. Briefly, an approximate location for the tumor was first selected based on magnetic resonance imaging (MRI), x-ray mammography and ultrasound images. In the selected area, the location of the maximum intensity of the optical attenuation coefficient at 786 nm was found (tumors have higher optical attenuation coefficients than normal tissues), and thereafter, a 3-D region was grown using the maximum intensity location as a seed with a cutoff at full-width at half-maximum. During this process, we examined the selected tumor region to determine whether the optical images provided tumor location and size consistent with the radiological imaging methods. Tumor values for each optically derived parameter are extracted by averaging each parameter over this entire tumor volume. Normal tissue values are derived by averaging overall normal tissues (i.e., excluding the tumor) in the same breast. The tumor-to-normal ratio of each parameter was thus calculated; the letter ‘r’ is added in front of the abbreviation of each parameter to indicate a relative (tumor-to-normal) value, for example, relative tissue oxygen saturation (rStO_2_), relative total hemoglobin (rTHC), relative oxyhemoglobin (rHbO_2_), relative deoxyhemoglobin (rHb) concentrations, and relative reduced-scattering coefficients (rμ_s_').

### Diffuse correlation spectroscopy (DCS) and relative mammary metabolic rate of oxygen (rMMRO_2_)

DCS was used to measure a blood flow index that, in turn, was used to calculate a tumor-to-normal ratio of mammary metabolic rate of oxygen of a subset of subjects (n = 6) who also had Ki67 analysis. The DCS measurements were performed on the same day as the DOT measurements. Details about the DCS instrument, the mathematical model and the input parameters have been described by Choe et al. [16]. Briefly, DCS measures a temporal intensity autocorrelation function of the detected light, and the decay rate of the autocorrelation function indicates blood flow, for example, faster decays correlate with faster blood flow. A tissue blood flow index, BFI, was derived from the measured DCS temporal light intensity autocorrelation functions by fitting the data to a solution of the correlation diffusion equation in the homogeneous semi-infinite geometry [1]. The DCS system employed a 786-nm-long coherence laser as a light source (delivered through a multi-mode fiber), a single-mode fiber was used to collect light and a fast-photon-counting avalanche photodiode was used for detection. A custom-built correlator board determined the normalized temporal intensity autocorrelation functions of the detected light.

For in vivo measurements, the tumor location in a supine position was identified either by palpation or by consulting radiology reports. Then a handheld probe with one source and one detector fiber 2.5 cm apart was gently placed on the breast tissue and data were collected from 10–12 points (1 cm apart) aligned on a line straddling the tumor center. The tumor and normal tissue points were averaged, respectively, to obtain one representative tumor and one normal region blood flow index for each subject. The relative blood flow (rBF) was calculated by taking the ratio between tumor and normal region BFI values.

Part of the analysis of this paper employed a mammary oxygen metabolism model that was introduced by Zhou et al. [13]. The tumor-to-normal ratio of the (relative) mammary metabolic rate of oxygen (rMMRO_2_) employed herein was slightly modified from the version introduced by Zhou et al. to monitor metabolic changes in breast tumors during neoadjuvant chemotherapy. rMMRO_2_ is expressed as follows:

$$ rMMR{O}_2=\frac{\gamma_N}{\gamma_T}\cdot \frac{rHb}{rTHC}\cdot rBF $$

where *γ* represents the percentage of blood volume contained in the venous component of the vascular system (i.e., StO_2_ = (1− γ)SaO_2_ + γ SvO_2_ where SaO_2_ and SvO_2_ are arterial and venous oxygen saturation, respectively [45]), the subscript *T* indicates tumor and *N* indicates normal tissue, and rBF is the tumor-to-normal ratio of BFI quantified by DCS [16]. Note, the so-defined rMMRO_2_ is closely related to the cerebral metabolic rate of oxygen extraction (CMRO_2_) parameter that has been studied extensively for metabolism measurements in the brain [1, 46–49].

### Microscopic analysis of tissue specimens

#### Slide selection and immunohistochemical analysis

Tissue blocks that include cancer tissues were reviewed, and blocks with the most cancer tissue were selected by a pathologist (MDF) for microscopic analysis of cancer properties. Immunohistochemical analysis of formalin-fixed paraffin-embedded tissue was performed using antibodies against Ki67 (Clone MIB-1; DAKO, Carpinteria, CA M7240; 1:20 dilution) and CD34 (Clone My10; BD Biosciences, San Jose, CA 347660; 1:80 dilution) on separate slides from the selected tissue block. Staining was done on a Leica Bond™ instrument using the Bond Polymer Refine Detection System (Leica Microsystems, Buffalo Grove, IL AR9800). Heat-induced epitope retrieval was required for Ki67 and this was performed for 20 minutes with ER1 solution (Leica Microsystems AR9961).

#### Digital analysis of the staining

**Fig. 2:**
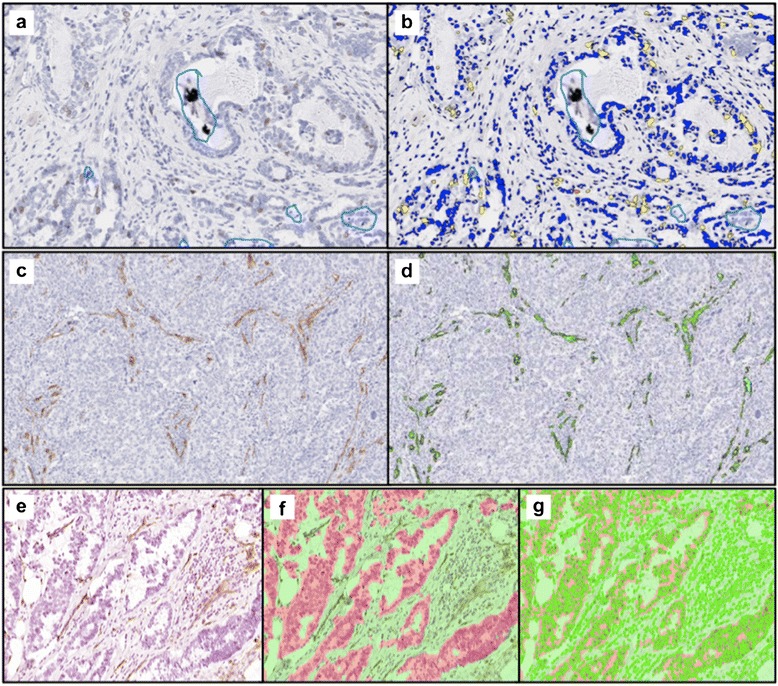
**a** Ki67-expresseing nuclei were stained by MIB-1 and appear as *brown spots*. Abnormal extreme dark spots caused errors in automated analyses and were excluded manually (see *blue loops*). **b** *Dark blue* regions designate cells in which the nuclei did not express Ki67, and *yellow* and *orange* regions designate cells with Ki67-expressing nuclei (note, *orange* signifies more intense expression). **c** CD34-stained vessels appear *brown*. **d** Detected vessels were highlighted in *neon green* using the Aperio algorithm. **e** The raw image was segmented into cancer (*pink*) versus non-cancerous (*green*) areas as shown in **f**. **g** Individual cells were detected as *neon green* loops. Based on nuclear shape, the compactness of the nuclei was calculated using inForm 1.0.2 (Perkin Elmer)

The stained slides were scanned using Aperio ScanScope (Aperio, Vista, CA, USA) with ×20 objective. In order to account for cancer heterogeneity, multiple regions of interest (ROI) were selected in the cancer and normal tissue samples (i.e., 2–7 ROI depending on the available amount of tissue and degree of heterogeneity) by SHC under the guidance of MDF and DM using the annotation tool of Aperio ImageScope, and MDF re-confirmed the selected ROI tissue types. We noticed that a few extremely dark spots appeared randomly on the slides, mostly clusters of the staining dye. These spots cause errors in the analysis, because the algorithm selects pixels with lower intensity than a threshold value as positive nuclei, and the dark spots have extremely low intensity values that can result in mistaken selection as strongly positive nuclei; thus, those areas were excluded manually using the annotation tool. An area-weighted average of the multiple ROI for each subject was used in the correlation study. The Ki67 and CD34 stained areas were differentiated from the non-stained areas by setting relative values of red-green-blue (RGB) optical densities (for defining the color of the stain) and the intensity of the defined color. These values were optimized by iterative adjustments: the detection algorithm with the set values was applied to multiple sample cases and the results were visually examined by MDF if the assigned color on the nuclei matched the actual features of the nuclei (Fig. 2). For Ki67 detection, the Ki67-expressing nuclei percent of the total number of nuclei in an ROI was calculated using the Aperio immunohistochemistry (IHC) nuclear image analysis algorithm (version 10.2). We counted 3278 nuclei per subject (on average) with a range of 270 to 4114 in the 15 subjects (Fig. 2a, b). Then, the tumor-to-normal ratio of Ki67-expressing nuclei was calculated to derive relative Ki67 values (i.e., rKi67). For this analysis, after subjects were selected and slides obtained, only normal glandular tissues with Ki67 expression (within each slide) were used.

For CD34-stained vessel properties, the Aperio microvessel analysis algorithm was used. An average of 118 vessels per ROI were detected (n = 19) and used for the quantification of micro-vessel density (number of vessels per unit area (μm^2^)), mean vessel and vascular area and the ratio of stained area to ROI area. Note, the mean vessel area includes the lumen in addition to the endothelial cells that form the vascular structure. To calculate the mean vessel/vascular area for each ROI, only vessels with a closed cross-section were automatically detected in the ROI first, then a mean value of the vessel/vascular area was calculated for each ROI (Fig. 2c, d).

For the nuclei morphology analysis, binary segmentation (cancer versus non-cancer) was used instead of the ROI selection. The digitized slides were converted to a multispectral data stack using Nuance 2.10.0 (Perkin Elmer). Then, using inForm 1.0.2 image analysis software (Perkin Elmer), tumor and normal tissues were segmented by the training algorithm, and the nuclear shapes were detected and used for the calculation of the nuclear compactness (Fig. 2e, f, g). Nuclear compactness is calculated using the following equation:

$$ \mathrm{Compactness}=4\pi \times \mathrm{Area}/{\mathrm{Perimeter}}^2. $$

### Statistical analysis

We used Pearson’s correlation coefficient (corr. coef.) to assess the linear association between the mean of the DOT physiological parameters and the microscopically assessed histopathological biomarkers. We generally report on possible linear relationships between the DOT physiological parameters and several biomarkers. We also used Spearman’s correlation coefficient to further explore the possibility of a monotonic, but non-linear relationship. The Wilcoxon ranked sum test was used to ascertain differences in DOT parameters in Ki67-positive and Ki67-negative groups (those defined by the 15 % cutoff threshold). The type I error rate was 0.05, and corrections were not made for multiple comparisons in this exploratory study.

## Results

### Association of DOT parameters with Ki67 cancer proliferation

**Fig. 3:**
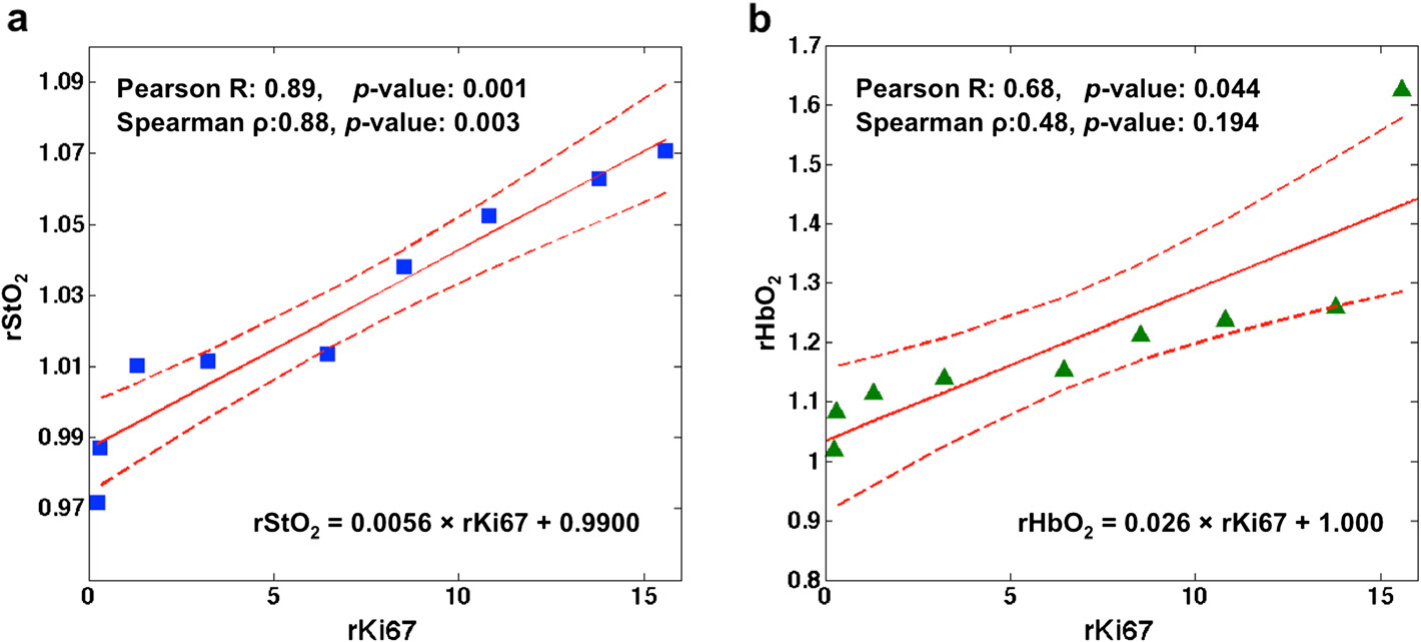
Correlation between **a** relative tissue oxygen saturation (rStO_2_) and rKi67 and **b** relative oxyhemoglobin concentration (rHbO_2_) and rKi67 (n = 9). Dotted lines indicate 95 % confidence interval for the mean of the linear fit. These pilot results suggest that more oxygen is present in the more proliferative cancer tissues

The percent range of the Ki67-nuclei used for determination of tumor-to-normal ratio of Ki67 (i.e., rKi67) varied from 0.05−23.45 % in cancer tissues (n = 9), and from 0.19−7.41 % in normal tissues (n = 9) (see Additional file 1: Table S2). More subjects were included for the DOT versus cancer-only Ki67-expression comparison study, and the Ki67 range for this latter investigation was 0.05−27.77 % in cancer tissues (n = 18). Among the DOT parameters, rStO_2_ and rHbO_2_ were linearly correlated with rKi67 as shown in Fig. 3 and Table 1 (Pearson correlation of 0.89, *p*-value 0.001 for rStO_2_, and Pearson correlation of 0.68, *p*-value 0.044 for rHbO_2_). Also, for cancer-only Ki67, rHb was inversely correlated with cancer Ki67 percent (Spearman correlation of −0.62, *p*-value 0.007; see Additional file 1: Table S3 for all correlation values between DOT physiological parameters and cancer Ki67 percent). The range of Ki67 values and those of other molecular biomarkers used in the remainder of the correlation studies are tabulated in Additional file 1: Table S4.

**Table 1:** Correlation between rKi67 and various relative DOT parameters

Analysis of correlation with rKi67 %	rStO_2_	rTHC	rHbO_2_	rHb	rμ_s_’
Pearson’s correlation coefficient	**0.89**	0.55	**0.68**	−0.33	0.44
*P*-value	**0.001**	0.126	**0.044**	0.393	0.24
Spearman’s correlation coefficient	**0.88**	0.40	0.48	−0.43	0.35
*P*-value	**0.003**	0.291	0.194	0.250	0.359

Relative tissue oxygen saturation (rStO_2_) and relative oxyhemoglobin concentration (rHbO_2_) were highly correlated with rKi67. Results with statistical significance (*p*-value <0.05) are shown in bold (n = 9). rTHC denotes relative total hemoglobin concentration, rHb denotes relative deoxyhemoglobin concentration, and rμ_s_' denotes relative reduced-scattering coefficients

**Fig. 4:**
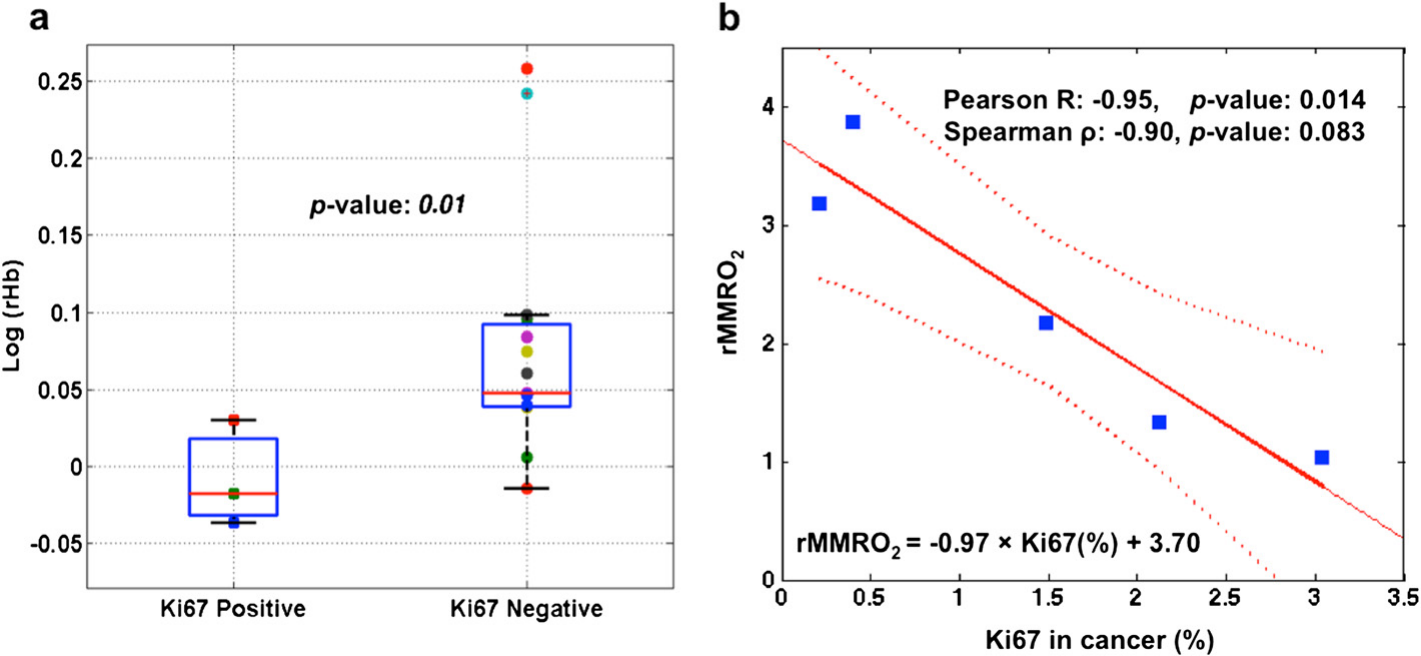
**a** Relative deoxyhemoglobin concentration (rHb) versus Ki67 expression in breast cancer tissues. Lower rHb is observed in Ki67-positive cancer compared to Ki67-negative cancer (*p*-value 0.01, n = 3 for Ki67-positive and n = 15 for Ki67-negative cancer). *Squares* and *circles* in the box plots show the values for each individual subject. On each box, the *central bar* is the median, the *edges* of the box mark the 25th and 75th percentiles and the *whiskers* extend to the most extreme data points not considered outliers. **b** Correlation between mammary metabolic rate of oxygen (rMMR_2_) and Ki67 in cancer for a subset of the Ki67-negative group (n = 5), for whom diffuse correlation spectroscopy flow measurements were available. *Dotted lines* indicate the 95 % confidence interval of the mean of the linear fit

Clinically, Ki67 expression is used as a biomarker for cancer proliferation. Tissue is termed Ki67-positive (or Ki67-negative) if the fraction of nuclei expressing Ki67 is above (or below) a specified cutoff, which is commonly set at 15 % [50–53]. In the present study, we tested whether DOT and DCS parameters are significantly different in more proliferative cancers compared to less proliferative cancers (i.e., as determined by the 15 % Ki67 cutoff). In this scenario, only rHb was found to differentiate Ki67-positive from Ki67-negative cancer, with lower values of rHb occurring for Ki67-positive cancer (*p*-value 0.01, Wilcoxon ranked sum test, Fig. 4a; see Additional file 1: Table S5 for values of all DOT physiological parameters of Ki67-positive and Ki67-negative cancer groups). Among the available subjects, 15 were Ki67-negative and 3 were Ki67-positive, but a *p*-value of 0.01 suggests the pilot result may be significant, even with the limited sample size.

In the comparison between rMMRO_2_ and Ki67 expression level, not all the subjects were measured with DCS: six subjects were available for analysis of the relationship between rMMRO_2_ and cancer-Ki67, and two subjects for analysis of the relationship between rMMRO_2_ and rKi67. Among the six subjects, five had low Ki67 values and belonged to the Ki67-negative group; the Ki67 percentage in these subjects exhibited significant inverse linear correlation with rMMRO_2_ (Pearson correlation −0.95, *p*-value 0.014, Fig. 4b). One subject belonged to the Ki67-positive group and did not follow the linear trend observed for the Ki67-negative group in Fig. 4b (the Ki67-positive subject is not shown). On analysis of the two subjects in whom rKi67 was compared to rMMRO_2_, higher rKi67 was associated with lower rMMRO_2_.

In addition, we ran predictability tests using the >15 % threshold to define Ki67-positive cancer. In this case, we found high negative predictive values (i.e., 100 %) for detection of non-proliferative cancer with specific thresholds for each of the DOT parameters. For example, if values of rHb <1.1 are considered proliferative, then 100 % of the negative predicted values are truly negative. However, given the small dataset (n = 18), the implications of this predictability result should not be overestimated. The main caveat is that only three subjects were in the Ki67-positive group (15 subjects were in the Ki67-negative group). Thus, it is difficult to ascertain the heterogeneity of the Ki67-positive group. Albeit imperfect, the strong correlation and predictability obtained from our DOT data suggest that DOT holds potential to predict proliferative status of breast cancer non-invasively and that the method is deserving of further exploration. This result can also aid the planning of a future study that will require more Ki67-positive samples.

### Association between DOT parameters and CD34 stained vascular properties

**Fig. 5:**
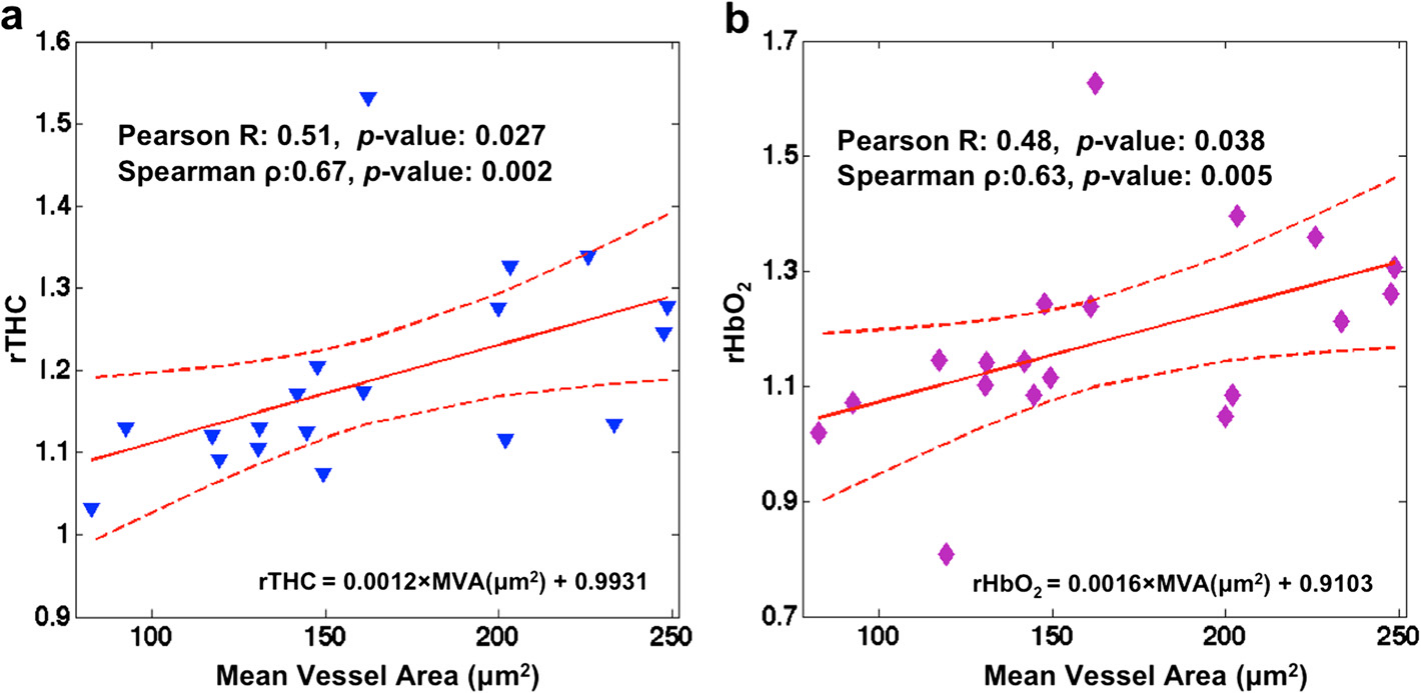
Correlation between **a** relative total hemoglobin concentration (rTHC) and **b** relative oxyhemoglobin concentration (rHbO_2_) versus mean vessel area (MVA, μm^2^) (n = 19). *Dotted lines* indicate the 95 % confidence interval of the mean of the linear fit. These pilot results suggest that diffuse optical tomography is measuring an increased blood supply in the larger-diameter blood vessels of these cancer tissues

Blood vessels in cancer tissues were detected and analyzed using the CD34 antibody. Among all the vascular properties accessible to this staining, i.e., micro-vessel density, mean vessel/vascular area, and ratio of stained area to analysis area, only mean vessel area (MVA) was significantly and linearly correlated with rTHC and rHbO_2_ (Pearson correlation coefficient of 0.51, *p*-value 0.027 and Pearson correlation of 0.48, *p*-value 0.038, respectively; see Fig. 5, Table 2). However, it should be noted that rTHC, rHbO_2_ and rμ_s_' had higher ranked monotonic correlation with MVA (Spearman correlation coefficient of 0.67, *p*-value 0.002; Spearman correlation of 0.63, *p*-value 0.005; and Spearman correlation coefficient of 0.52, *p*-value 0.024, respectively).

**Table 2:** Correlation between CD34-stained mean vessel areas (MVA) and DOT parameters

Analysis of corrlelation with MVA (μm^2^)	rStO_2_	rTHC	rHbO_2_	rHb	rμ_s_’
Pearson’s correlation coefficient	0.26	**0.51**	**0.48**	−0.10	0.32
*P*-value	0.291	**0.027**	**0.038**	0.684	0.182
Spearman’s correlation coefficient	0.45	**0.67**	**0.63**	−0.22	**0.52**
*P*-value	0.057	**0.002**	**0.005**	0.362	**0.024**

MVA was correlated with relative total hemoglobin concentration (rTHC), relative oxyhemoglobin concentration (rHbO_2_) and relative reduced-scattering coefficients (rμ_s_’) (n = 19). Results with statistical significance (*p*-value <0.05) are shown in bold. *DOT* denotes diffuse optical tomography, rStO_2_ denotes relative tissue oxygen saturation, and rHb denotes relative deoxyhemoglobin concentration

### Association of DOT parameter with nuclear morphological analysis

On nuclear morphology analysis there was weak positive correlation between rHbO_2_ and the tumor-to-normal ratio of nuclear compactness (Pearson correlation coefficient of 0.43, *p*-value 0.049, n = 21; see Additional file 1: Table S6 for values for correlation between all DOT physiological parameters and relative nuclear compactness.). Additionally, rStO_2_ was weakly positively correlated with cancer nuclear compactness (Spearman correlation coefficient of 0.47, *p*-value 0.034; see Additional file 1: Table S7 for values for correlation between all DOT physiological parameters and relative nuclear compactness.). When the nucleus is spherical, then the compactness is unity. In our case, all of the measured compactness values were smaller than unity.

As the tumor grade worsened (i.e., became higher), although a slight trend of increasing rStO_2_ and decreasing rHb was observed, it was not statistically significant (n = 29). Lastly, distinct differentiation of DOT parameters was not observed in any of the estrogen receptor (ER)-, progesterone receptor (PR)- and human epidermal growth factor-2 (HER2)-positive versus -negative groups (n = 31).

### Differences between breast cancer subtypes

All but one of the samples used for the rKi67 correlation analysis were luminal A subtype; the other sample was basal-like and its rKi67 value was the median. For Ki67 (not rKi67) in cancer tissues, within the luminal A subtype (n = 12), negative correlation was observed between Ki67 percent and rHb (Spearman correlation of −0.57) with a trend towards statistical significance (*p*-value 0.059). No trend was observed within the basal-like subtype (n = 4). Correlation was not analyzed separately in two subjects with HER2-positive cancer due to small sample size.

In the CD34 comparison studies, within the basal-like subtype group (n = 4), the Pearson correlation coefficient between MVA and rTHC was 0.96 (*p*-value 0.045). Within the luminal A group (n = 12), several statistically significant correlations were observed between MVA and rTHC (Spearman correlation of 0.69, *p*-value 0.016), rHbO_2_ (Spearman correlation of 0.71, *p*-value 0.012) and rμ_s_’ (Spearman correlation of 0.62, *p*-value 0.035). Two subjects with HER2-positive and one with the luminal B subtype were not included in the subtype correlation analysis.

In the nuclear compactness comparison study, within subjects with the basal-like subtype (n = 5), there was evidence of positive correlation between rμ_s_' and the nuclear compactness of cancer cells (Pearson correlation of 0.87) with a trend towards statistical significance (*p*-value 0.057). No correlation was observed within the luminal A subtypes (n = 13). Two subjects with HER2-positive cancer and one with the luminal B subtype were not included in the subtype correlation analysis.

In addition, among the group that included subjects only with DOT images and subtype information (no tissue slides, n = 32), we found that basal-like (n = 6) and HER2-positive (n = 3) subtypes were significantly differentiated (*p*-value 0.023) by rTHC values, although the sample size was very small.

## Discussion

Our study revealed that the tumor-to-normal (relative) ratio of Ki67-positive nuclei is correlated positively with rStO_2_ measured by DOT. This observation suggests that the more proliferative cells reside in more oxygenated environments, i.e., more oxygenated compared to surrounding normal tissues. Further, the percentage of Ki67 nuclei in cancer-only tissues was inversely correlated with DOT-measured rHb, and in a subset of subjects with low Ki67 expression, an inverse correlation between Ki67 expression level and rMMRO_2_ [13] was observed (*R* = −0.95, *p*-value 0.014). We found that MVA correlated positively with DOT-measured relative total hemoglobin and oxyhemoglobin concentration; this observation suggests that an increased blood supply to the tumors is due to increased blood vessel size rather than blood vessel density. The combination of these pilot results suggests that more proliferative cancer cells demand less oxygen. Finally, we observed that in less oxygenated environments, cell nuclei tend to exhibit a more elongated shape. Overall, the results of the pilot study corroborate expectations that macroscopic measurements of breast cancer physiology using DOT can reveal information about the microscopic pathological properties of breast cancer and hold potential to complement pathological biomarker information.

The Ki67 proliferation biomarker was chosen because it is one of the biomarkers of choice for determination of cancer severity [32, 54]. Baseline measurements of Ki67 have correctly predicted patient prognosis [32, 55, 56], and high levels of Ki67 predict better responses to chemotherapy [31, 57, 58]. The percentage ranges of the Ki67-nuclei of our tissue samples are consistent with reported values in the literature, including those obtained from automated evaluation methods [59, 60]. Specifically, in two studies, more than 72 % of 379 patients [59] and 50 % [61] of 3658 patients had cancer with <15 % Ki67 expression, and in a study by Konsti et al., the mean Ki67 expression level in 1334 subjects was 8.8 % [60]. By contrast, Ki67 expression level in normal breast tissues was found to be less than 3 % in several studies [32, 62–64]. We observed that rKi67 correlated positively with rStO_2_ and rHbO_2_. Our finding of correlation between rStO_2_ and rKi67 is supported by the observations of Ueda et al. [30] who measured higher tissue oxygenation in subjects with complete pathological response (i.e., compared to those with incomplete pathological response) prior to neoadjuvant chemotherapy. In our study, patients with higher levels of tissue oxygenation exhibited a high level of Ki67, and the combination of all reports supports the well-known finding that a high Ki67 level predicts better response to neoadjuvant chemotherapy [31, 57, 58]. Ki67 is known to be correlated with apoptosis [32, 65, 66]. One of our previous works showed simultaneous increase of tissue oxygenation and temperature caused by an uncoupled apoptotic state at the beginning of neoadjuvant chemotherapy in a subject who was found to have complete pathological response [19]. The strong correlation between Ki67 and tissue oxygenation found in this current work combined with the previous result supports the correlation between Ki67 and apoptosis in breast cancer.

We also found that rHb was inversely correlated with cancer-only Ki67, and that rHb was lower in Ki67-positive cancer compared to Ki67-negative cancer (using the 15 % cutoff, note this cutoff can vary from 10−20 % in different clinics). It is noteworthy that in the tumors with rHb smaller than unity (i.e., less deoxyhemoglobin in the tumor than surrounding normal tissues), the rStO_2_ was larger than unity (higher tissue oxygenation in the tumor compared to normal tissues): this finding suggests that in more proliferative tumors, oxygenated blood is supplied to the tumor but the excess oxygen is not fully used.

Importantly, we took an additional step to further investigate the relationship between proliferation and oxygen metabolism. Specifically, we calculated the tumor-to-normal rMMRO_2_ in a subset of the data; only a small number of subjects among the population were studied in this retrospective analysis because only a small number were measured with DCS, which provides the relative blood flow (rBF) data needed to calculate rMMRO_2_. In the tumors with very low Ki67 expression, there was inverse correlation between oxygen metabolism and Ki67 level. In total, the full set of Ki67/DOT-parameter comparison studies suggest that higher tissue oxygenation tends to arise in cancers with higher expression of Ki67, lower deoxyhemoglobin concentration is often present in more proliferative cancers, and tumors with rHb <1 have rStO_2_ >1. Although more blood is supplied to the cancer compared to normal tissue (i.e., all subjects had rTHC >1), the level of oxygenated hemoglobin in the tumor remains high, and less oxygen is utilized for cancer metabolism (yielding a lower tumor rHb).

These preliminary conclusions about the relationship between proliferation and oxygen metabolism in breast cancer can be explained by the Warburg effect [44], which accounts for the fact that some cancer cells go through glycolysis to increase biomass without using oxygen, despite sufficient oxygen in the tissue environment [67, 68]. While these findings are interesting, we note that our model for rMMRO_2_ has several simplifying assumptions about the vasculature and tissue heterogeneity [1], and the DCS and DOT measurements utilized different measurement geometries. In the future, it would be interesting to include measurement of Myc, a glycolysis marker, which would enable further exploration of the relationship between cancer metabolism and proliferation [69].

Finally, we note that in the rKi67/DOT correlation study, all of the samples with Ki67 expression in normal tissues were obtained from premenopausal patients. Therefore, it is possible that the premenopausal status of all subjects used for the rKi67/DOT correlation could have contributed to the correlations observed. Greater proliferation in normal or benign tissues has been observed in premenopausal women [70]. Ultimately, when more tissues become available, we will be able to ascertain the importance of menopausal status.

CD34-stained vascular properties were also compared to DOT parameters. CD34 is expressed in the endothelial cells of micro-vessels that migrate during angiogenesis [33, 71, 72], and CD34 was used to assess vascularity in the tumor samples. In the samples studied, only tumor areas were studied so that only tumor vascularity was assessed. Distant normal tissue was not available for investigation of the patient biopsies. We chose CD34 instead of CD31, because a prior investigation [72] compared various monoclonal antibodies for angiogenesis assessment and found that the antibody to CD34 was more reliable than the CD31 antibody. Among compared vascular parameters, MVA in cancer tissues was correlated with rTHC, rHbO_2_ and rμ_s_'. These results met our expectation that DOT measures increased blood supply to cancer through the abnormally dilated blood vessels in cancer tissues [73].

The DOT tumor-to-normal tissue parameters have previously been shown by Choe and co-workers [6] to exhibit excellent differentiation between benign and malignant lesions. However, the association between DOT parameters and tumor pathology was not explored. Here, the use of tumor-to-normal ratios for both optical and histological data minimized the effects of inter-subject absolute property variation. Optical measurement of total hemoglobin concentration has been compared with histologically evaluated vascular properties in other studies. In a study by Srinivasan et al. using near-infrared tomography, a correlation value of 0.3 was obtained (*p*-value not reported) for association between total hemoglobin concentration and the vessel density in 12 subjects, including both benign and malignant tumors [38]. Zhu et al. measured total hemoglobin concentration using a combined system of ultrasound and near infrared optical imaging, observing correlation between total hemoglobin concentration and micro-vessel density (MVD, R = 0.64, *p*-value <0.05, 10 samples from 6 subjects) [39]. In a study of neoadjuvant chemotherapy responders, Pakalniskis and colleagues observed significant correlation between CD105-stained MVD and total hemoglobin concentration in subjects with complete pathological response, but not in those with partial pathological response (*p* <0.001, n = 7, correlation coefficient not reported) [37]. In our study, vessel area was more strongly correlated (Spearman correlation coefficient of 0.67, *p*-value 0.002, n = 19) with total hemoglobin concentration than the MVD, which was not significantly correlated with total hemoglobin concentration. We note that generally the various studies reported in the literature differ in the antibodies employed, the analysis methods utilized (automatic vessel detection versus manual hotspot counting), and, in some cases, in the way parameters are defined. For example, in our work, MVD was defined as the number of vessels per unit area (μm^2^), but in the work of Pakalniskis et al., MVD is defined as a percentage of the combined areas of CD105-stained vessels per total area of the slide.

Nuclear morphology has been shown to exhibit a more elongated shape in cancer cells than normal cells [34–36]. Hajihashemi et al. observed higher μ_s_' and more elongation in cancer nuclei than the normal or benign tissues [34, 35]. In our study, the tumor-to-normal ratio of nuclear compactness was smaller than 1, which indicates that the tumor had more elongated nuclei than the normal tissues. The relative and cancer nuclear compactness was only weakly correlated with rHbO_2_ and rStO_2_, respectively, suggesting that in a less oxygenated environment, the nuclei tend to be more elongated.

Looking forward, tissue samples from normal breast tissue need to be collected. The correlation between rTHC and the CD34-stained MVA could be augmented and improved, for example, if normal vessels were available. Although our statistical power to detect clinically relevant associations is limited by the number of samples available for analysis, the cohort we have investigated has yielded some potentially important findings. Further studies will be needed to confirm the results reported here. In future studies, tissues sent out for the trial, the Investigation of Serial studies to Predict Your therapeutic response with imaging and molecular analysis (I-SPY, American College of Radiology and Imaging Network Protocol 6657), will be also included. Finally, it is noteworthy that inclusion of multiple ROI in each specimen produced stronger correlation by partially accounting for cancer heterogeneity.

## Conclusion

This pilot study has shown that DOT-measured and DCS-measured breast cancer physiological parameters are correlated with proliferation of cancer cells and with formation of abnormally dilated blood vessels in cancer. The results suggest that DOT may be useful as a non-invasive assessment tool for specific cancer biomarkers, especially for cancer proliferation. As Ki67 is a well-known prognostic index and an early predictor of tumor response to chemotherapy, the high correlation between Ki67 and DOT-measured tissue oxygenation further suggests the potential of optical imaging and monitoring. The optical methods utilize non-ionizing radiation and can be easily employed at the bedside. These features, coupled with predictive capability, are particularly attractive for patients who need frequent monitoring, for example, during chemotherapy, and for women with radiographically dense breast tissue.

## Additional file

Additional file 1: Table S1. Subjects for the correlation studies between diffuse optical tomography (DOT) parameters versus various cancer biomarkers. **Table S2.** Cancer and normal Ki67 values used for the calculation of tumor-to-normal ratio of Ki67. **Table S3.** Correlation between cancer Ki67 percent versus DOT-relative parameters. **Table S4.** Range of values of the biomarkers used for the correlation studies. **Table S5.** DOT parameters for the Ki67-positive and Ki67-negative cancer groups. **Table S6.** Correlation of relative nuclear compactness versus DOT parameters. **Table S7.** Correlation of cancer nuclear compactness versus DOT parameters. DOT three-dimensional (3-D) image reconstruction steps.

## Abbreviations

3-D: three-dimensional
AD: Angela DeMichele
AGY: Arjun G. Yodh
BFI: tissue blood flow index
BJC: Brian Czerniekcki
CCD: charge-coupled-device
CMRO_2_: cerebral metabolic rate of oxygen extraction
corr. coef.: correlation coefficient
CW: continuous-wave
DCS: diffuse correlation spectroscopy
DM: Daniel Marinez
DOT: diffuse optical tomography
DRB: David R. Busch
ER: estrogen receptor
FD: frequency-domain
FDG: fluorodeoxyglucose
HER2: human epidermal growth factor receptor 2
HK: Helen Kim
IHC: immunohistochemical analysis
I-SPY: Investigation of serial studies to predict your therapeutic response with imaging and molecular analysis
JT: Julia Tchou
MAR: Mark A. Rosen
MDF: Michael D. Feldman
MDS: Mitchell D. Schnall
MEP: Mary E. Putt
MVA: mean vessel area
MVD: mean vessel density
NAC: neoadjuvant chemotherapy
NHLBI: National Heart Lung and Blood Institute
NIH: National Institute of Health
PR: progesterone receptor
rBF: relative blood flow
RC: Regine Choe
RGB: red-green-blue
rHb: relative deoxyhemoglobin concentration
rHbO_2_: relative oxyhemoglobin concentration
rKi67: tumor-to-normal ratio of Ki67, or relative Ki67
rMMRO_2_: tumor-to-normal ratio of mammary metabolic rate of oxygen, or relative mammary metabolic rate of oxygen
ROI: region(s) of interest
rStO_2_: relative tissue oxygen saturation
rTHC: relative total hemoglobin concentration
rμ_s_’: relative reduced-scattering coefficients
SaO_2_: arterial oxygen saturation
SHC: So Hyun Chung
StO_2_: tissue oxygen saturation
SvO_2_: venous oxygen saturation
