# Supplementary material for: Macroscopic optical physiological parameters correlate with microscopic proliferation and vessel area breast cancer signatures
Source: Breast Cancer Res. 2015 May 27;17:72. doi: 10.1186/s13058-015-0578-z (PMC4487833; doi:10.1186/s13058-015-0578-z)
Supplement: Additional file 1: Table S1. — Subjects for the correlation studies between diffuse optical tomography (DOT) parameters versus various cancer biomarkers. Table S2. Cancer and normal Ki67 values used for the calculation of tumor-to-normal ratio of Ki67. Table S3. Correlation between cancer Ki67 percent versus DOT-relative parameters. Table S4. Range of values of the biomarkers used for the correlation studies. Table S5. DOT parameters for the Ki67-positive and Ki67-negative cancer groups. Table S6. Correlation of relative nuclear compactness versus DOT parameters. Table S7. Correlation of cancer nuclear compactness versus DOT parameters. DOT three-dimensional (3-D) image reconstruction steps. [file 13058_2015_578_MOESM1_ESM.doc]

Table s1 Subjects for the correlation studies between DOT parameters versus various cancer biomarkers. (ER: estrogen receptor, PR: progesterone receptor, HER2: human epidermal growth factor receptor 2)

| **Biomarkers** | **N** | **Age**  **(Years)** | **Pre-**  **menopause** | **Post-**  **menopause** | **BMI**  **(kg/m^2^)** | **Lesion Size**  **(cm, the longest)** |
| --- | --- | --- | --- | --- | --- | --- |
| Ki67 ratio of cancer to normal tissues | 9 | 40±6 | 9 | 0 | 26.9±8.3 | 2.5±1.0 |
| Ki67 in cancer | 18 | 48±10 | 10 | 8 | 27.4±7.0 | 2.3±1.1 |
| CD34 in cancer | 19 | 49±10 | 10 | 9 | 28.1±7.2 | 2.1±1.0 |
| Nuclear compactness | 21 | 48±10 | 11 | 10 | 27.9±6.8 | 2.3±1.1 |
| Tumor grade and hormonal/genetic types | 32 | 48±9 | 18 | 14 | 27.7±6.4 | 2.3±1.3 |

Table s2 Cancer and normal Ki67 values used for the calculation of tumor-to-normal ratio of Ki67 (i.e. rKi67). Increments between each rKi67 is also shown.

| **Cancer Ki67** | **Normal Ki67** | **rKi67** | **Increments** |
| --- | --- | --- | --- |
| 0.05 | 0.21 | 0.25 |  |
| 2.12 | 7.41 | 0.29 | 0.04 |
| 0.36 | 0.28 | 1.29 | 1.01 |
| 6.38 | 1.99 | 3.21 | 1.91 |
| 16.66 | 2.58 | 6.46 | 3.26 |
| 23.45 | 2.75 | 8.52 | 2.06 |
| 7.70 | 0.71 | 10.81 | 2.29 |
| 10.59 | 0.77 | 13.80 | 2.99 |
| 3.04 | 0.19 | 15.59 | 1.79 |

Table s3 Correlation between cancer Ki67% versus DOT relative parameters. rHb inversely correlated with cancer Ki67%. The result with statistical significance (*p*-value<0.05) is shown in bold (N=18).

| Cancer Ki67% versus | rStO_2_ | rTHC | rHbO_2_ | rHb | rµ_s_' |
| --- | --- | --- | --- | --- | --- |
| Pearson's corr. coef. | 0.29 | -0.08 | 0.1 | -0.42 | -0.22 |
| *p*-value | 0.246 | 0.766 | 0.681 | 0.080 | 0.387 |
| Spearman's corr. coef. | 0.49 | 0.03 | 0.2 | **-0.62** | -0.2 |
| *p*-value | 0.1 | 0.915 | 0.427 | **0.007** | 0.436 |

Table s4 Range of values of the biomarkers used for the correlation studies.

| **Biomarkers** | **Minimum** | **Maximum** |
| --- | --- | --- |
| Tumor-to-normal ratio of Ki67 (N=9) | 0.25 | 15.59 |
| Ki67 in cancer used for the rKi67 (N=9, %) | 0.05 | 23.45 |
| Ki67 in cancer (N=18, %) | 0.05 | 27.77 |
| Vessel area assessed by CD34 staining in cancer (N=19, μm^2^) | 82.47 | 261.68 |
| Tumor-to-normal ratio of nuclear compactness (N=21) | 0.77 | 1.05 |
| Nuclear compactness in all cancer (N=21, no unit) | 0.48 | 0.67 |
| Nuclear compactness in triple negative cancer (N=4, no unit) | 0.48 | 0.54 |

Table s5 DOT parameters for the Ki67-positive and Ki67-negative cancer groups. Notice, rHb is lower in Ki67-positive cancers with statistical significance. Std. stands for standard deviation. The result with statistical significance (*p*-value<0.05) is shown in bold.

| **Properties** | **Status** | **n** | **rStO_2_** | | | **rTHC** | | | **rHbO_2_** | | | **rHb** | | | **rµ_s_'** | | |
| --- | --- | --- | --- | --- | --- | --- | --- | --- | --- | --- | --- | --- | --- | --- | --- | --- | --- |
|  |  |  | Mean | Std. | *p*-value | Mean | Std. | *p*-value | Mean | Std. | *p*-value | Mean | Std. | *p*-value | Mean | Std. | *p*-value |
| cancer Ki67 % | >15% | 3(17%) | 1.03 | 0.01 | 0.427 | 1.16 | 0.10 | 1 | 1.19 | 0.1 | 0.574 | 0.98 | 0.08 | **0.010** | 1.35 | 0.17 | 0.302 |
|  | ≤15% | 15(83%) | 0.99 | 0.09 |  | 1.19 | 0.12 |  | 1.17 | 0.18 |  | 1.21 | 0.24 |  | 1.57 | 0.37 |  |

Table s6 Correlation of Relative nuclear compactness versus DOT parameters. rHbO_2_ was weakly correlated with relative nuclear compactness, which indicates that with less oxygenated-hemoglobin, nuclei are more elongated (N=21). The result with statistical significance (*p*-value<0.05) is shown in bold.

| Relative Nuclear compactness vs. | rStO_2_ | rTHC | rHbO_2_ | rHb | rµ_s_' |
| --- | --- | --- | --- | --- | --- |
| Pearson's corr. coef. | 0.38 | 0.32 | **0.43** | -0.32 | 0.13 |
| *p*-value | 0.088 | 0.151 | **0.049** | 0.156 | 0.564 |
| Spearman's corr. coef. | 0.33 | 0.11 | 0.22 | -0.35 | -0.08 |
| *p*-value | 0.144 | 0.633 | 0.329 | 0.12 | 0.737 |

Table s7 Correlation of cancer nuclear compactness versus DOT parameters. rStO_2_ was correlated with cancer nuclear compactness, which also indicates that in less oxygenated environments, the nuclei are more elongated (N=21). The result with statistical significance (*p*-value<0.05) is shown in bold.

| Cancer nuclear compactness vs. | rStO_2_ | rTHC | rHbO_2_ | rHb | rµ_s_' |
| --- | --- | --- | --- | --- | --- |
| Pearson's corr. coef. | 0.34 | 0.25 | 0.37 | -0.27 | 0.16 |
| *p*-value | 0.126 | 0.265 | 0.095 | 0.228 | 0.479 |
| Spearman's corr. coef. | **0.47** | 0.17 | 0.41 | -0.28 | 0.19 |
| *p*-value | **0.034** | 0.449 | 0.07 | 0.226 | 0.412 |

***DOT 3-D Image Reconstruction steps***

Average optical properties derived from the frequency domain measurements were used as initial guesses for the reconstructions. A multi-spectral version [1, 2] of the standard tomography approach based on the photon-diffusion-equation [3-5] was employed for the reconstructions. Specifically, a finite-element method based numerical solver [6] calculated the fluence rate at each detector position, and this calculation was compared to the measured fluence rate. To suppress image artifacts associated with sources and detectors, a non-uniform, unstructured mesh with higher nodal concentrations at source/detector planes was developed and utilized for the finite-element computations. We defined a Rytov-type objective function χ^2^ [7, 8], with Intralipid/ink fluid reference measurements used for the normalization. The unknowns (chromophore concentrations and reduced-scattering coefficient) were then updated using an iterative conjugate-gradient-based scheme [9] that was modified to include data at all wavelengths simultaneously. A memory-efficient conjugate gradient method permitted use of large data sets (4 x 10^4^ spatial × 6 spectral data). Furthermore, parallel computation was implemented to speed up reconstruction time. As results, three-dimensional images of oxy-, deoxy- and total-hemoglobin concentrations, tissue oxygenation and reduced-scattering coefficients were obtained and tumor-to-normal ratio of the physiological information was calculated.

**References for Supplementary Data**

1. Corlu A, Choe R, Durduran T, Lee K, Schweiger M, Arridge SR, Hillman EMC, Yodh AG: **Diffuse optical tomography with spectral constraints and wavelength optimization**. *Applied Optics* 2005, **44**(11):2082-2093.

2. Durduran T, Choe R, Culver JP, Zubkov L, Holboke MJ, Giammarco J, Chance B, Yodh AG: **Bulk optical properties of healthy female breast tissue**. *Physics in Medicine and Biology* 2002, **47**(16):2847-2861.

3. Durduran T, Choe R, Baker WB, Yodh AG: **Diffuse optics for tissue monitoring and tomography**. *Reports on Progress in Physics* 2010, **73**(7):1-43.

4. Furutsu K: **On the diffusion equation derived from the space-time transport equation**. *J Opt Soc Am A* 1980, **70**:360-366.

5. Groenhuis RAJ, Ferwerda HA, Tenbosch JJ: **Scattering and Absorption of Turbid Materials Determined from Reflection Measurements .1. Theory**. *Applied Optics* 1983, **22**(16):2456-2462.

6. Arridge SR, Schweiger M: **Photon-Measurement Density-Functions .2. Finite-Element-Method Calculations**. *Applied Optics* 1995, **34**(34):8026-8037.

7. Choe R, Corlu A, Lee K, Durduran T, Konecky SD, Grosicka-Koptyra M, Arridge SR, Czerniecki BJ, Fraker DL, DeMichele A, Chance B, Rosen MA, Yodh AG: **Diffuse optical tomography of breast cancer during neoadjuvant chemotherapy: A case study with comparison to MRI**. *Medical Physics* 2005, **32**(4):1128-1139.

8. Choe R, Konecky SD, Corlu A, Lee K, Durduran T, Busch DR, Czerniecki BJ, Tchou JC, Fraker DL, DeMichele A, Arridge SR, Schweiger M, Culver JP, Rosen MA, Schnall MD, Chance B, Yodh AG: **Differentiation of Benign and Malignant Breast Tumors by In-Vivo Three-Dimensional Parallel-Plate Diffuse Optical Tomography**. *Journal of Biomedical Optics* 2009, **14**(2):024020.

9. Arridge SR, Schweiger M: **A gradient-based optimisation scheme for optical tomography**. *Optics Express* 1998, **2**(6):213-226.
